# Editorial: Research ethics and integrity in the artificial intelligence era

**DOI:** 10.3389/frma.2026.1931866

**Published:** 2026-08-05

**Authors:** Josiline Chigwada, Patrick Ngulube

**Affiliations:** SIRGS, University of South Africa, Pretoria, South Africa

**Keywords:** artificial intelligence, editorial, publishing, research ethics, research integrity

## Introduction

Artificial intelligence (AI) is reshaping academic publishing, offering efficiency, speed, and novel tools to streamline processes (Carobene et al., 2023; Chubb et al., 2022; Fiorillo and Mehta, 2024). However, its growing presence in manuscript preparation, submission, review, and distribution introduces complex ethical questions (Bouhouita-Guermech et al., 2023; Kocak, 2024; Miao et al., 2024; Resnik and Hosseini, 2025). As the boundaries between human effort and machine input blur, maintaining integrity, transparency, and fairness becomes a challenge. This introduction identifies key ethical concerns in each phase of the publishing life cycle, proposing strategies for navigating these issues while preserving the trustworthiness of academic knowledge production. The publishing life cycle is a structured process that involves several stages through which a piece of research or a manuscript passes from conception to final publication (PLOS, 2024). Each stage poses unique ethical challenges, particularly with the increasing use of AI.

Research and manuscript preparation include idea generation where the research idea is conceptualized and the researcher develops hypotheses, conducts experiments, or explores theoretical frameworks to contribute new knowledge to the field. If the data involves empirical work, data is collected, analyzed, and interpreted. A manuscript detailing the research findings, methodologies, literature review, and discussions is drafted. After submission, the manuscript would be reviewed by the chief editor to check if it fits the journal's scope, standards, and guidelines. If the manuscript passes the screening, the editor sends it to peer reviewers for in-depth evaluation. If it does not meet the requirements, it may be desk-rejected at this stage. The editor selects experts in the relevant field to evaluate the manuscript, and the peer reviewers assess the originality, quality, significance, and soundness of the research. Reviewers provide detailed feedback, including suggestions for improvements or corrections. Once the peer review process is complete and revisions have been made, the editor makes a final decision, and if accepted, the manuscript is sent to a copyeditor who checks for grammatical errors, consistency, style adherence, and clarity. The article is then published, and the post-publication stage is when the article is promoted to maximize its reach. Throughout the publishing life cycle, there are ethical considerations that should be dealt with, and these have been worsened by using AI in academic writing (Chetwynd, 2024; Hosseini et al., 2023; Miao et al., 2024).

### Idea generation and research

Idea generation and research involve the conception of a research idea, designing the study, and gathering data. AI tools can assist with literature review, data analysis, and even predicting research trends (Bolaños et al., 2024). Natural language processing (NLP) models can generate hypotheses or review massive amounts of literature to aid in formulating research questions. The ethical issues include plagiarism and idea ownership, bias in data sets, and integrity of hypotheses (Akgun and Greenhow, 2022; Khalifa and Albadawy, 2024). AI-generated content or ideas may blur the lines between what is original work by the researcher and what is sourced from AI tools. Ownership of AI-generated ideas is a gray area. AI systems may inadvertently introduce or exacerbate biases in the data used for research, leading to skewed findings. There's a risk that AI could be used to generate hypotheses without appropriate human judgment, possibly resulting in weak or unsubstantiated research questions. In this Research Topic, Abubakar and Adan interrogate how user experience mediates, and disciplinary context moderates, perceived research integrity when AI is used at the ideation and inquiry stage.

### Manuscript preparation

This stage includes drafting, writing, and formatting the manuscript. AI tools have become ubiquitous in the preparation of research manuscripts, assisting with grammar, data analysis, and even content generation (Carobene et al., 2023). While these technologies can improve the quality and accessibility of scholarly writing, they raise significant ethical questions. AI tools, like grammar and style checkers (e.g., Grammarly), citation managers, and even automated summarization systems, are often used during the writing process. Some more advanced tools can generate entire sections of text. Issues such as authorship attribution, ghostwriting, authorship misconduct, and misleading use of AI arise (Al-kfairy et al., 2024). If AI is substantially involved in the writing process, the question of who gets credit as an author becomes complex. Is the AI a tool or a co-author? Some argue that AI cannot hold authorship, while others raise concerns about the fairness of not attributing significant AI input. AI-generated text could be passed off as the work of human authors, raising concerns about transparency and academic honesty. Researchers may misrepresent the extent to which AI contributed to the writing, which could deceive peer reviewers and readers (Al-kfairy et al., 2024). To what extent should AI-generated content be disclosed in manuscripts? Should AI be credited as a co-author, or should it remain a background tool? Failure to identify the role of AI could lead to accusations of plagiarism or unethical authorship practices (Carobene et al., 2023). The evolving role of AI in manuscript preparation raises questions about the transparency of authorship. Journals may need to establish clearer guidelines for disclosing the use of AI tools in research and writing. Failure to do so risks undermining trust in the scholarly record, as readers and reviewers may not fully understand the extent of AI's involvement in the production of knowledge. This editorial proposes a three-tier disclosure framework which are:

(a) Tier 1—language and mechanical assistance such as grammar proofreading, spell-check, style polishing, reference formatting. This can be equivalent to a professional copy editor. A brief acknowledgment in the methods or acknowledgment section is sufficient since there are no co-authorship implications, or methodological disclosure required.

(b) Tier 2—Drafting assistance such generation of paragraph-level text, summarization of prior literature, rewording of author-supplied content, and code scaffolding. This requires a statement naming the tool and version, the sections affected, the prompts used, and confirmation that authors verified every generated claim and citation. Authors remain fully accountable for accuracy and originality.

(c) Tier 3—View derivation and analytical contribution such hypothesis generation, interpretation of results, synthesis of arguments, thematic coding, or model-driven inferences that shapes the paper's conclusions. This requires a dedicated AI contribution statement describing the analytical role, the data or prompts used, the human oversight applied, and a reproducibility appendix with prompts, model version, dates and key outputs.

This tiered approach preserves the practical usefulness of AI at Tier 1 while imposing proportionate transparency at Tiers 2 and 3, and it aligns with Malik et al.'s global insights on ChatGPT's influence on academic writing and plagiarism policy, and with Chigwada and Ngulube's analysis of publisher author-guidelines in this Research Topic.

### Peer review

In this phase, the manuscript is evaluated by experts in the field for quality, accuracy, and contribution to the discipline. AI has begun to play a role in peer review, promising to streamline the process by identifying relevant reviewers, detecting plagiarism, and even providing automated evaluations of manuscripts (Bauchner and Rivara, 2024; Carobene et al., 2023). However, integrating AI into peer review introduces several ethical challenges such as bias and fairness in review, transparency in AI-assisted review, and automation bias (Fiorillo and Mehta, 2024). AI-driven recommendation systems for selecting peer reviewers raise concerns about bias and fairness (Checco et al., 2021; Hosseini and Resnik, 2025). Algorithms may inadvertently favor certain reviewers based on citation patterns, affiliations, or geographical proximity, potentially marginalizing scholars from underrepresented groups or institutions. This could reinforce existing disparities in academic publishing and compromise the fairness of the review process. The use of AI in the peer review process raises questions about transparency. Reviewers may not disclose that AI was used to help form their critiques, which could affect trust in the process. Reviewers might place undue trust in AI's evaluations, leading to errors being overlooked or valid work being unfairly criticized based on AI-driven analysis (Al-kfairy et al., 2024). These algorithmic fairness concerns are given rare empirical texture in this Research Topic by Pasipamire and Muroyiwa, whose examination of algorithm bias in AI that publishing inequities are not incidental but structurally reproduced byopaque recommender and evaluation systems. Tsekea and Mandoga's Zimbabwean university library study, and Salani and Tapfuma's account of AI in the African book sector, further situate these fairness risks within the infrastructural realities of the Global South.

Some journals have experimented with using AI to generate peer-review reports or assist reviewers by highlighting sections of the manuscript for scrutiny (Checco et al., 2021; Lin et al., 2023). While this can reduce reviewer workload, it raises ethical questions about the quality of the reviews produced. AI cannot understand the interpretations or the ethical implications of certain research methodologies, which are often critical to a thorough review. AI tools used to detect plagiarism and self-plagiarism in submissions rely on vast databases of published work (Page et al., 2023). However, these tools may inadvertently flag legitimate citations or overlaps due to algorithmic limitations, potentially harming authors' reputations unjustly. Moreover, the integration of these tools with proprietary databases raises concerns about the handling of intellectual property and the ethical use of authors' manuscripts. AI is also influencing the decision-making processes in academic publishing, from recommending acceptance or rejection of manuscripts to ranking papers based on their impact (Perkins and Roe, 2024). While these systems offer efficiency, they also introduce ethical risks. As AI becomes more prominent in reviewing and editorial decision-making, there is a risk of diminishing the role of human judgment in scholarly publishing. AI lacks the ethical reasoning, creativity, and subjectivity that human editors and reviewers bring to the process.

### Manuscript revision

Authors revise their manuscripts based on the feedback from reviewers. AI tools can offer suggestions on how to rewrite sections for clarity or suggest additional references and content. The ethical issues that come into play are plagiarism concerns and excessive dependence on AI (Zhai et al., 2024). AI-driven suggestions may pull information from uncredited sources, and over-reliance on AI tools for revision may result in the loss of the author's voice or human oversight in the research process (Zhai et al., 2024). Once a manuscript is accepted, it undergoes editing for grammar, consistency, and formatting. AI-based grammar checkers and style tools are commonly used to improve the readability of the manuscript. Human oversight is an ethical issue where even if AI systems can proofread or edit more efficiently, their inability to fully grasp context or meaning might lead to errors being introduced or some content being mishandled (Darwin et al., 2024). Dahal's contribution to this Research Topic on adapting qualitative-research quality criteria in the era of generative AI, is particularly instructive here, as revision is precisely where generative outputs are most likely to displace reflective authorial judgement.

### Publication and dissemination

Editors may rely on AI systems to help decide which manuscripts are suitable for publication based on automated evaluations of the manuscript's content or predicted impact (Carobene et al., 2023; Khalifa and Albadawy, 2024; Lin et al., 2023). These systems can introduce ethical issues related to algorithmic bias, potentially privileging certain types of research or methodologies that align with past patterns of successful publications. Such biases may undermine diversity in Research Topics and approaches, distorting the scientific record. Furthermore, AI systems that predict a manuscript's future impact based on citation trends and social media mentions raise ethical concerns regarding the commercialization of academic publishing (Wiwanitmkit and Wiwanitkit, 2024). Research that is expected to generate more attention might be favored over less popular but equally significant studies. When the final manuscript is published and made available to the public, AI can be used for automatic indexing, metadata creation, and even personalized content recommendations for readers. Misinformation, copyright and licensing, and dissemination bias can be prevalent where AI can be exploited to generate fake research papers, misleading readers and damaging the integrity of scientific knowledge (Hamed et al., 2024; Májovský et al., 2023). Issues arise concerning who owns the copyright for AI-generated content, and whether AI should be recognized as a contributor. AI-driven recommendation algorithms can prioritize certain types of research over others, potentially marginalizing minority voices or less trendy topics (Deldjoo et al., 2024). Sridharan and Sivaramakrishnan's systematic review of retractions in AI literature in this Research Topic, offers a concrete empirical anchor for these dissemination-stage risks.

### Post-publication monitoring

After publication, AI tools can be used to track citations, reader engagement, and potential retractions. Tools like plagiarism detection software or AI systems to identify problematic papers, for example, those with ethical concerns or fraudulent data, are used post-publication (Elkhatat et al., 2023; Miao et al., 2024). Issues such as surveillance and privacy, retraction, and correction come into play. The use of AI to monitor the spread and influence of published papers could raise concerns about privacy and the surveillance of researchers' work (Heidt, 2024). If AI identifies errors or fraud post-publication, the process for retracting or correcting the work may be unclear, particularly if human error is mixed with AI-driven analysis. AI-driven recommendation engines, used by academic databases and journal platforms to suggest related articles or authors, may inadvertently amplify already well-cited works, contributing to the Matthew effect where the rich get richer. This could marginalize lesser-known or emerging scholars, particularly those from underrepresented regions or fields, thereby reinforcing existing academic hierarchies. Moreover, AI tools used for indexing manuscripts and managing metadata must be carefully monitored to avoid errors or misclassifications that could affect the accessibility and discoverability of research. For instance, improper keyword assignment or misclassification could exclude certain works from being included in key academic databases or alter the way they are perceived in search results. Pinzon et al. analysis of AI-powered fraud and the erosion of online survey integrity, together with Alsharefeen and Al Sayari's case study of faculty perspectives on academic integrity policy, highlights that post-publication monitoring is inseparable from the pre-publication integrity architecture. Yusuf et al.'s study of librarians' AI competency, and Salhab's investigation of AI in higher education instruction, remond us that monitoring capacity rests on the human infrastructures that surround the technology.

## Conclusion

AI's integration into the publishing life cycle offers efficiencies and improvements but also introduces significant ethical issues that challenge the integrity of research, the fairness of review processes, and the authenticity of authorship. Transparency, clear guidelines, and continuous oversight are essential to mitigate these risks and ensure that AI enhances rather than undermines academic publishing. As shown across the papers, this Research Topic documents the ethical issues surrounding the use of AI in scholarly publishing. It demonstrates the need for all the stakeholders in the publishing life cycle to take heed of the research ethics and integrity issues in the AI era. This is because, AI is transforming the academic publishing landscape, introducing both opportunities for innovation and significant ethical challenges. Ensuring AI tools are used responsibly throughout the publishing life cycle is crucial for maintaining research integrity. Ethical frameworks addressing bias, transparency, accountability, and access must evolve alongside these technological advancements. Therefore, the academic community can preserve the values of fairness, inclusivity, and integrity in scholarly publishing by fostering a balanced relationship between AI and human judgment.

In order to move governance from the conceptual to implementation, there are some red lines which must not be crossed by each stakeholder. Authors should not submit AI-generated content, results, citations, images as if human produced. In addition, they should not list AI systems as authors, do not use AI to fabricate, manipulate, or reinterpret findings, and should not upload confidential, unpublished third party or personally identifiable data to public AI tools. Authors should declare all AI use and state clearly that they take full responsibility for accuracy, originality, and integrity. Reviewers should not upload manuscripts, figures or supplementary data into external generative AI systems, delegate the substantive review judgement to AI, nor let AI draft reviews that misrepresent the reviewer's own reading of the work. Editors should not base the acceptance or rejection of a manuscript on AI-generated evaluations or use AI-recommender systems. Publishing institutions should not deploy AI systems for screening, reviewer matching, or decision support without documented bias, security, and data governance assessments. They should not monitize author or reviewer data through AI training without informed consent, nor permit AI-generated content in the version of a record without editorial and author accountability. Taken together, these stakeholder-specific obligation translate principles to transparency, accountability, fairness and human oversight into operations, which this Research Topic calls for.
